# Cardiovascular profiles associated with white matter hyperintensities in healthy young women

**DOI:** 10.3389/fphys.2022.979899

**Published:** 2023-01-13

**Authors:** Carole A. McBride, Zane Russom, Ella Achenbach, Ira M. Bernstein, Julie A. Dumas

**Affiliations:** ^1^ Department of Obstetrics, Gynecology and Reproductive Sciences, University of Vermont Larner College of Medicine, Burlington, VT, United States; ^2^ Department of Psychiatry, University of Vermont Larner College of Medicine, Burlington, VT, United States

**Keywords:** cardiovascular, preeclampsia, white matter hyper intensities, MRI, neurovascular aging, hypertension, vascular compliance

## Abstract

Women who experience hypertension in pregnancy have increased risk of both chronic hypertension and dementia. High blood pressure is associated with increased evidence of white matter hyperintensities (WMH) in brain imaging. WMH are disruptions of the white matter of the brain that occur with demyelination and axonal degeneration, are associated with vascular disease, occur more frequently in people with hypertension, and are associated with cognitive impairment. We evaluated the relationship between WMH and subclinical cardiovascular function in healthy young nulliparous women and women with a history of early-onset preeclampsia. Sixty-two reproductive-aged women were assessed during the follicular phase of the menstrual cycle after a 3-day sodium/potassium-controlled diet. Half of participants had a history of early-onset preeclampsia, and half were nulliparous. Blood was drawn to assess inflammatory markers. Cardiovascular assessments included tonometric blood pressure monitoring, volume loading to assess vascular compliance, echocardiography to assess cardiac ejection time, brachial pulse wave velocity of the brachial artery, assessing cardiovascular stiffness, and brachial artery flow mediated vasodilation to assess endothelial mediated dilatory response. T2 fluid-attenuated inversion recovery (FLAIR) MRI imaging was obtained. Two raters, blinded to cardiovascular assessments and pregnancy history, reviewed MRI scans for evidence of WMH using the Fazekas rating scale. WMHs were detected in 17 women; 45 had normal white matter structure. Participants with Fazekas score>0 had exaggerated response to volume loading compared to women with a Fazekas score of 0 and longer cardiac ejection times. Fazekas scores >0 had lower brachial flow-mediated vasodilation and increased white blood count compared to those with no evidence of WMH. Women with WMH had reduced cardiovascular compliance, and a trend towards decreased endothelial responsiveness compared to those without WMH. These data demonstrated that the relationship between cardiovascular and brain health was detectable in young, healthy, reproductive-aged women, and may play a role in later development of clinical disease. These findings may help identify women who are at risk for cognitive decline and pathological aging.

## Introduction

A history of hypertension in pregnancy, including preeclampsia, is a recognized risk factor for adverse cardiovascular outcomes in women (Ray et al., 2005; Mongraw-Chaffin et al., 2010). Hypertension in pregnancy is defined as new onset hypertension during pregnancy. Preeclampsia develops after 20-week gestation and is characterized by new-onset hypertension with evidence of end organ damage. It is well established that women who develop hypertension in pregnancy have increased incidence of cardiovascular morbidity in later life (Anderson, 2007; Bellamy et al., 2007; D'Agostino et al., 2013; Garovic et al., 2010; Honigberg et al., 2019). They are also at greater risk of later-life cognitive impairment, including vascular dementia and Alzheimer’s disease (Postma et al., 2013; Postma et al., 2014; Wiegman et al., 2014; Abheiden et al., 2015; Mielke et al., 2016; Theilen et al., 2016; Fields et al., 2017; Fox et al., 2018; Adank et al., 2021).

White matter hyperintensities (WMH) are similarly considered to be risk factors for adverse cardiovascular and neurovascular aging, including as predictors of faster decline in global cognitive performance, increased risk of cerebrovascular events, including stroke, dementia, and death, and as a predictor of Alzheimer’s disease (Debette and Markus, 2010; Mortamais et al., 2014; Wardlaw et al., 2015). WMH are commonly associated with hypertension and small vessel disease (Camerino et al., 2021; Wartolowska and Webb, 2021).

In young adults, WMH are relatively uncommon and usually appear as punctate lesions when they are detected (Fazekas et al., 1987; Wahlund et al., 2001). Additionally, WMHs have been identified as occurring more frequently in women with a history of hypertension in pregnancy when compared to age-matched women with a history of normotensive pregnancy (Wiegman et al., 2014; Postma et al., 2016).

Studies evaluating WMH in healthy young women are few, despite the recognized value of identification as an early risk factor for later life morbidity. The co-occurrence of cardiovascular risk factors in the development of hypertension in pregnancy, cardiovascular disease, and dementia raises questions about the mechanisms behind vascular contributions to cognitive decline and dementia. We sought to identify associations between measures of cardiovascular risk and the presence of white matter hyperintensities in the brains of healthy young women, hypothesizing that phenotypes of subclinical cardiovascular function would be associated with the presence of WMHs in healthy young women.

## Materials and methods

**FIGURE 1:**
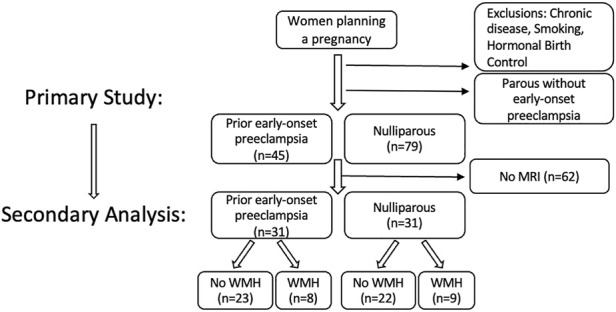
Flow diagram of study inclusion/exclusion of the primary study and secondary analysis presented here, to evaluate whether there are cardiovascular associations between the presence/absence of white matter hyperintensities identified in the brains of healthy reproductive-aged women. I.

This study was a secondary analysis of participants recruited through open advertisement prior to planned pregnancy. Study inclusion is presented in Figure 1. Participants had no history of chronic medical conditions, including hypertension, diabetes, autoimmune disease, or other known chronic medical conditions. Sixty-two young, healthy women from the primary cohort received magnetic resonance imaging (MRI) and were included in this secondary analytic cohort. Of the sixty-four, thirty-one were nulliparous and thirty-one had a history of early-onset preeclampsia but were not hypertensive at the time of assessment. Participant inclusion was solely based on the availability of MRI data, and it was by chance that the groups were of equal size. All participants were non-smokers and were not using hormonal contraceptives or intrauterine devices at the time of evaluation. This study was approved by the University of Vermont Institutional Review Board and the separate University of Vermont Clinical Research Center Scientific Advisory Committee in 2011 (Study 10–226). Written consent was obtained from all participants prior to enrollment, and participants were compensated for their time.

Evaluations were conducted from 2011 to 2015. Study visits were timed to occur during the follicular phase of the menstrual cycle, and measurements were made over the course of a single day. Participants were provided with 3-day of a sodium/potassium balanced diet prepared by a registered dietitian to minimize dietary-related hemodynamic variation. Assessments were made in the morning, following an overnight fast. They were asked to abstain from anti-inflammatory medications and decongestants for 48 h prior, and alcohol and caffeine for 24 h prior to evaluation. Blood was drawn for a complete blood count, comprehensive metabolic panel, lipid panel, and insulin levels. Insulin resistance was calculated using the formula for Homeostatic Model Assessment for Insulin Resistance (HOMA-IR) (Matthews et al., 1985).

Cardiovascular evaluations included assessment of cardiac output, cardiac ejection time, and pulse wave velocity using Doppler ultrasound and simultaneous electrocardiogram (Lewis et al., 1984). Cardiac ejection time and aorto-femoral velocity were evaluated using dual Doppler ultrasound and EKG, using time from EKG R wave to peak systolic flow in the aortic root (Bernstein et al., 2016). Similarly, pulse wave velocities (PWV) were calculated from EKG R wave to peak systolic flow in the popliteal and brachial arteries and corrected for height and cardiac ejection time. These assessments are methods of evaluating cardiac and peripheral arterial stiffness. Pulse wave velocity is considered the gold standard for assessing arterial stiffness (O'Rourke, 1990; Najjar et al., 2008).

Body composition was measured using dual-energy X-ray absorptiometry (DEXA) using a GE Lunar Prodigy Encore scanner.

Flow mediated vasodilation was assessed using previously reported techniques (Morris et al., 2015). Briefly, blood flow in the brachial and popliteal arteries, individually, was restricted using a blood pressure cuff. The restriction remained for 5-min, after which Doppler ultrasound was used to collect cine clips of vascular response for 90 s following release of vasoconstriction. Maximum dilation change was calculated from baseline. This test assessed endothelial function as arterial dilation is driven by the release of nitric oxide from the endothelium.

Blood pressure was measured tonometrically to determine beat-to-beat changes in systolic and diastolic blood pressure, pulse pressure, and mean arterial pressure using the Finopres Pro (Enschede, Netherlands). Baseline values were assessed, as well as the response to a volume challenge. A 500 mL bolus of Lactated Ringer’s saline was administered over approximately 10-min to evaluate vascular compliance and left ventricular diastolic function (Fujimoto et al., 2013; Bernstein et al., 2016). Tonometric changes were assessed prior to, during, and for 15-min post-infusion. Area under the curve for infusion and post-infusion monitoring was calculated as an overall measure of response to infusion for changes in blood pressure, pulse, and cardiac output (Bernstein et al., 2016).

**FIGURE 2:**
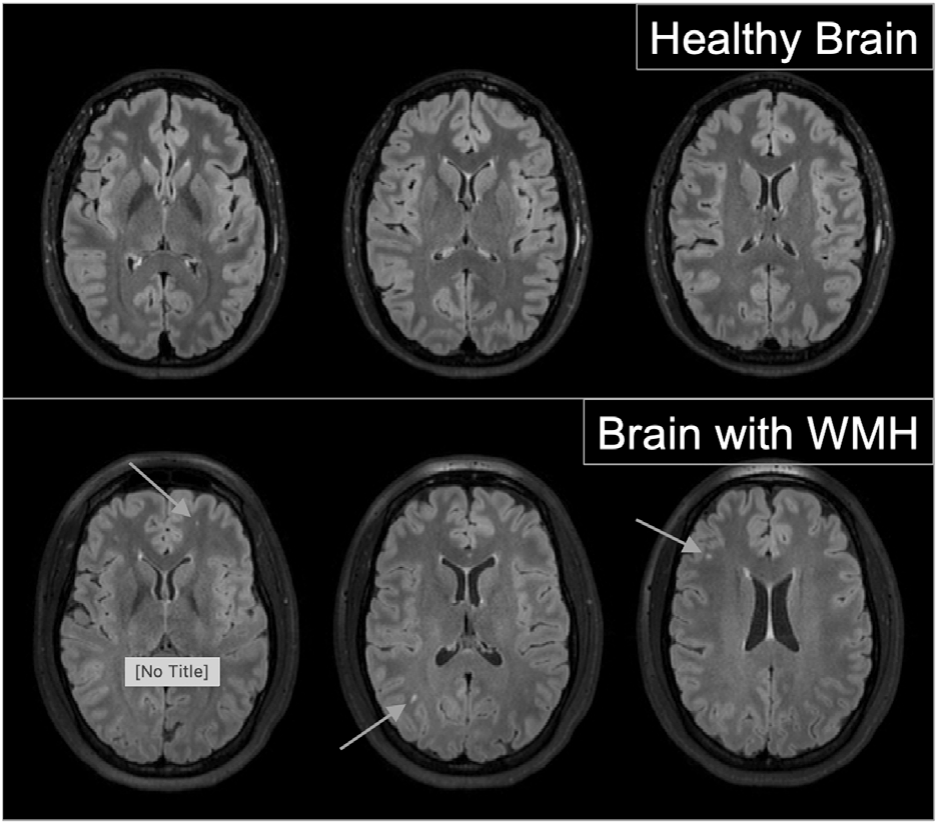
Examples of healthy brain images (top panel) and images from brains with evidence of which matter hyperitensities (bottom panel) from fluid-attenuated Inversion Recovery (FLAIR) magnetic resonance imaging.

Brain MRI images were obtained using a 3-T Philips research magnet at the MRI Center for Biomedical Imaging at the University of Vermont. T2 Sagittal Fluid-Attenuated Inversion Recovery (FLAIR) images were obtained with 1.0 mm isotropic resolution to assess white matter lesions. High resolution T1-and T2-weighted images were used to rule out incidental pathology by a neuroradiologist. White Matter Hyperintensities (WMH) were assessed using the Fazekas scale on T2-weighted FLAIR images (Fazekas et al., 2002). WMH are identified as focal lesions in the white matter of the brain, detected in MRI T2 fluid-attenuated inversion recovery (FLAIR) imaging. Weighted T2 imaging reduces the ventricular signal, making cerebro-spinal fluid appear black and WMHs appear as a bright spot. The Fazekas score is a rating system for the presence and severity of WMHs, with quantification including healthy with no WMH, grade 1 with punctiform lesions, grade 2 early confluent lesions, and grade 3 MWH appearing more diffuse (Fazekas et al., 1987). Example images are presented in Figure 2. These images were assessed by two adjudicators, blinded to patient history and cardiovascular measures, for the presence of WMHs and scored according to the Fazekas score. While initial agreement occurred with greater than 80% of participants, when discrepancies occurred, a third experienced MRI technologist, also blinded, reviewed the images. As these women were young and healthy, the majority of those with WMH were Fazekas score 1, which is punctiform in nature. Due to this, we then dichotomized the findings for analyses, looking at the presence or absence of WMHs.

Cardiovascular and biochemical assessments were chosen based on *a priori* hypotheses that features of poor cardiovascular health, including higher cardiac output, higher blood pressure, faster cardiac eject time (indicating a stiffer myocardium), a greater response to a volume challenge (indicating poor compliance), a lower flow-mediated vasodilatory response (indicating decreased endothelial function), and faster pulse wave velocity (indicating stiffer arterial vessels) are associated with poor cardiovascular outcomes and that these would influence brain health through identification of WMHs. As our primary objective was to identify whether there were detectable cardiovascular differences between participants with and without evidence of WMH, we compared baseline characteristics, physiologic measures, and biochemical analyses between those with and without WMH using Students t-tests or Mann-Whitney for continuous variables and Chi-squared tests for categorical variables. Satterthwaite corrections were used where data was non-normally distributed. Rates of WMHs were also compared between women with and without a history of early-onset preeclampsia to determine if a history of preeclampsia increased the frequency of WMHs. Statistical significance was determined based on *p* < 0.05, and all statistical analyses were performed using SAS statistical software version 9.4 (SAS Institute, Cary, NC).

## Results

Participants were mean age 31 ± 5 years, with body mass index 26 ± 6 kg/m^2^, and were predominantly (88%) Caucasian. Half of participants (31) were nulliparous, and half had a history of early-onset preeclampsia (31). Of those with a history of prior early-onset preeclampsia, 25 were primiparous, and 6 had experienced early-onset preeclampsia in 2 or more pregnancies. Forty-five women did not have evidence of WMH, and seventeen had evidence of WMH with Fazekas score 1. No women had evidence of WMH loads greater than Fazekas score 1. There were no differences in the rate of WMH when comparing women with a history of preeclampsia to nulliparas. Out of the prior preeclamptics, 8 out of 31 women assessed had evidence of WMHs while 9 out of 31 nulliparous women had detected WMHs. The interval between time from prior delivery for the prior preeclamptics ranged from 9 months to 6.5 years, with a mean interval of 1.5 years for those with WMH and 2.6 years for those with no WMH. There were no demographic differences between women with and without WMH, including between age, BMI, race, or history of preeclampsia. Comparisons between women with and without WMH are presented, with demographics and body composition (Table 1), and cardiovascular and biochemical (Table 2) assessments outlined. No differences were detected in body composition, lipid profiles, hematocrit, C-reactive protein, or insulin resistance.

**TABLE 1:** Comparisons of demographic, physical, and anthropomorphic observations between women with and without evidence of white matter hyperintensities. Data are presented as mean ± standard deviation or frequency.

Characteristics of participants	No WMH (n = 45)	WMH (n = 17)	*p*-value
Age, years	31 ± 5	31 ± 5	90
Race, % Caucasian	84%	88%	71
History of Preeclampsia	51%	47%	77
Cycle Day, days	9.6 ± 3.9	11.3 ± 3.1	09
Body Composition			
Height, cm	163 ± 6.9	165 ± 7.1	55
Weight, kg	68.7 ± 16.5	73.5 ± 19.1	32
BMI, kg/m^2^	25.7 ± 5.7	27.1 ± 6.6	40
Fat Body Mass, %	38 ± 9	39 ± 10	62
Android Fat Mass, %	42 ± 12	42 ± 12	90

**TABLE 2:** Comparisons of cardiovascular and biochemical observations between women with and without evidence of white matter hyperintensities. Data are presented as mean ± standard deviation or frequency.

Characteristics of participants	No WMH (n = 45)	WMH (n = 17)<	*p*-value
Cardiovascular comparisons			
Mean Arterial Pressure, mmHg	90 ± 10	91 ± 9	84
Response to Volume Challenge, Liters Cardiac Output/min	6.6 ± 11.6	15.7 ± 14.3	01
Cardiac Ejection Time, sec	0.147 ± 0.02	0.160 ± 0.02	02
Brachial Pulse Wave Velocity, m/sec	11.7 ± 4.2	18.0 ± 16.4	12
Popliteal Pulse Wave Velocity, m/sec	3.9 ± 0.7	3.7 ± 0.6	26
Popliteal Flow-mediated Vasodilation, %	8.3 ± 7.3	4.6 ± 6.2	07
Cardiac Output, L/min	4.6 ± 1.0	4.6 ± 1.0	86
Biochemical Comparisons			
White Blood Cell Concentration, k/mL	5.6 ± 1.3	6.3 ± 1.6	06
Hematocrit, %	35.9 ± 2.9	36.4 ± 2.6	56
Triglycerides	85 ± 42	112 ± 96	28
HDL, mg/dL	49.5 ± 9.7	48.9 ± 10.7	86
Total Cholesterol, mg/dL	158.6 ± 25.8	164.6 ± 7.9	47
Triglycerides, mg/dL	84.6 ± 41.6	111.8 ± 96.4	28
C-reactive Protein, mg/mL	6.0 ± 7.2	5.5 ± 6.6	83
Insulin Resistance, HOMA-IR	1.36 ± 0.91	1.71 ± 1.34	34

**FIGURE 3:**
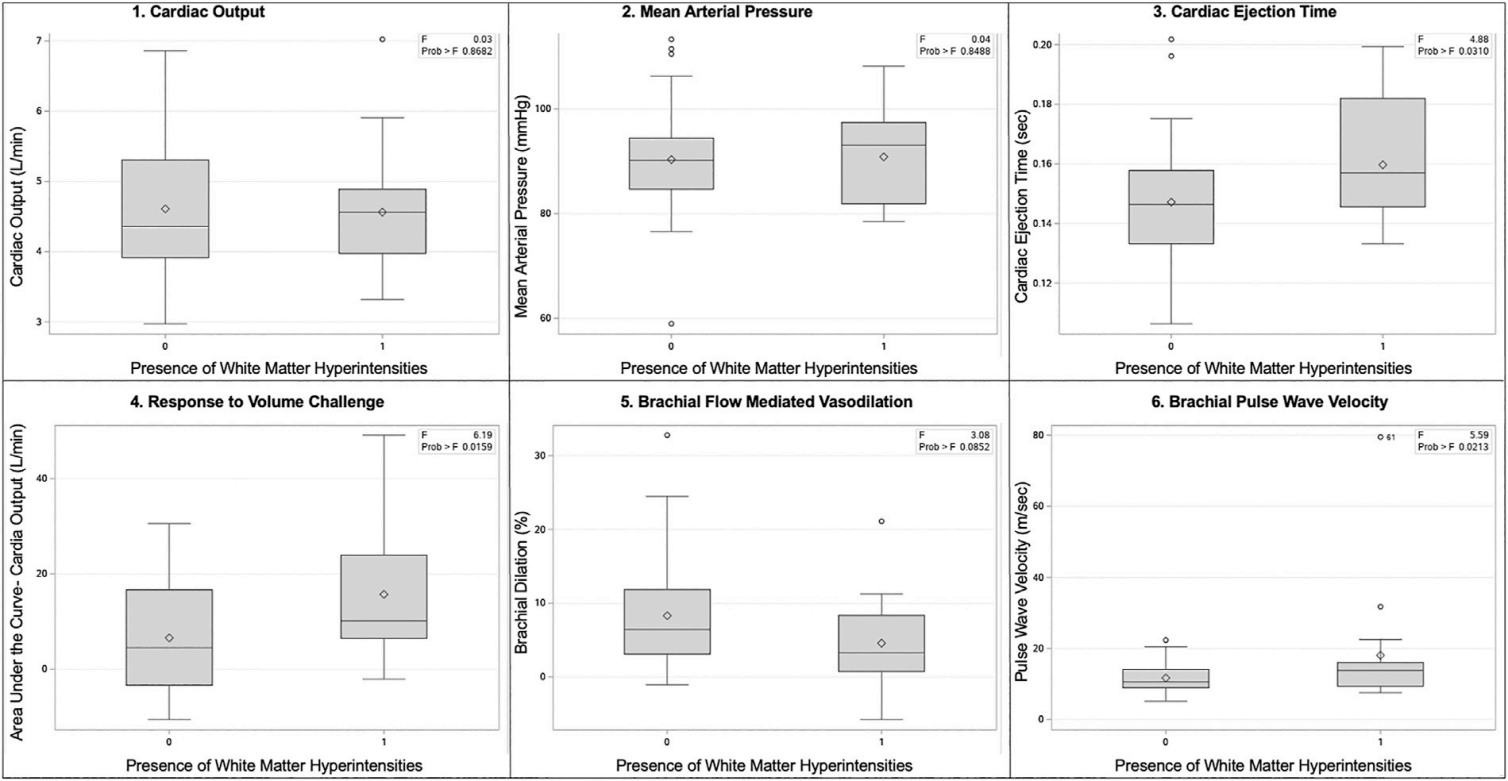
Box and whisker plots of select physiologic comparisons based on the presence or absence of white matter hyperintensities. Panels are as follows: 1) cardiac output, 2) mean arterial pressure, 3) cardiac ejection time, 4) response to volume challenge, 5) brachial flow mediated vasodilation, 6) brachial pulse wave velocity.

Physiologic assessments are presented in Figure 3. In both groups, mean systolic blood pressure was 121 mmHg and mean diastolic was 70 mmHg. There were no differences in cardiac output, blood pressure, or pulse wave velocity. We observed an exaggerated response in cardiac output to volume loading, (No WMH = 6.6 ± 11.6 vs. WMH = 15.7 ± 14.3 L/min, *p* = 0.01) and increased cardiac ejection time (no WMH = 0.147 ± 0.020 vs. WMH = 0.160 ± 0.020 s, *p* = 0.02) in women with WMHs compared to those without. While not powered to detect statistical significance, we also observed elevated white cell count (No WMH = 5.6 ± 1.3 k.mL vs WMH = 6.3 ± 1.6, *p* = 0.056), indicative of enhanced inflammation, and a smaller response to flow-mediated vasodilation in both the popliteal (No WMH = 8.3% ± 1.2% dilation vs WMH = 4.6 ± 1.5, *p* = 0.07) artery.

## Discussion

We observed that healthy young women with WMH had identifiable subclinical cardiovascular markers of altered cardiovascular function compared to similar women without white matter lesions. These markers included a slower cardiac ejection time and larger increase in cardiac output in response to volume challenge, suggesting reduced cardiovascular capacity. While we expected to observe faster pulse wave velocity in those with WMH, indicative of arterial stiffness, our healthy young population did not support this hypothesis. Although there is a great deal of evidence that women who have a history of preeclampsia are more likely to develop hypertension, and hypertension is a risk factor for WMH, we detected no increase in WMH frequency in women with a history of early-onset preeclampsia compared to nulliparous women in our relatively small study sample.

Studies evaluating the interplay between cardiovascular function and WMHs in a cohort of healthy young women are few. In one study of young adults, increased aortic PWV was associated with decreased gray matter volume and the development of WMH (Bown et al., 2021). In another study, women aged 20–50 were evaluated for presence of WMH in the setting of migraine. In migraine sufferers, WMH volume was positively associated with elevated central systolic blood pressure and faster carotid-femoral pulse wave velocity (Cheng et al., 2018). These findings in a similar population based on age distribution also identified relationships between WMHs and altered cardiovascular function. This supports the hypothesis that alterations in the cerebral small vessels, areas of inflammation, and disruptions of the blood-brain barrier associated with WMH may be vascular in origin (Moroni et al., 2020).

Due to a limited number of studies evaluating WMH in reproductive-aged women, there is debate over WMH onset and their persistence in women with preeclampsia. Soma-Pillay et al. (2017). longitudinally evaluated women with severe preeclampsia at delivery, 6-month, and 1-year postpartum. Out of 94 women included in the study, 62% had WMH at delivery. By 1-year postpartum, 48% of women who had been diagnosed with preeclampsia still had WMHs, a finding that was driven by a 5-fold increase in women who continued to require antihypertensives. Within our study sample, the mean interval from most recent delivery for the women with a history of prior preeclampsia was 2.2 years, though those with WMH were at mean interval 1.5 years compared to women without WMHs who were 2.6 years from their preeclamptic deliveries. Studies of WMHs several years following preeclampsia-affected pregnancies report a continuation of the increased frequency in women who have had preeclampsia compared to those with normotensive pregnancy. At 5-year postpartum, 37% of prior preeclamptic women had evidence of WMHs, compared to 21.3% of normotensive women (Wiegman et al., 2014). A similar evaluation at mean 6-year postpartum by Postma et al. (2016) compared women who had experienced early-onset preeclampsia or eclampsia to women who experienced normotensive pregnancies. In this study, the authors observed 40% of women with a history of early-onset preeclampsia had detectable WMH while 21% of controls had detectable lesions. This differs from our findings, where similar numbers of participants in each group had detectable WMHs.

Detection of WMHs in reproductive-aged women raises questions about long term trajectories of neurovascular decline. WMH increase in frequency and severity with aging, with frequency increasing after middle-age. Longitudinal evaluations have demonstrated the predictive nature of WMH on later adverse outcomes, with participants with WMH at baseline having a 14% elevated risk of cognitive impairment and all-cause dementia, a 25% increased risk of Alzheimer’s disease and 73% increased risk of vascular dementia (Hu et al., 2021). Longitudinal evaluation of middle-aged populations with WMH have demonstrated increased WMH volume to be associated with increased cognitive decline and decreased IQ in adulthood (d'Arbeloff et al., 2019). Women with a history of gestational hypertension performed poorly on word learning cognitive testing, including delayed and immediate recall approximately 15 years after pregnancy when compared to women with normotensive pregnancies (Adank et al., 2021). Differences in structural neuroanatomy have been reported in women with a history of preeclampsia, including reduced cortical grey matter, decreased temporal lobe white matter microstructure, increased temporal lobe white matter lesion volume, and altered microstructural integrity compared to women with no history of hypertension in pregnancy (Mielke et al., 2016; Siepmann et al., 2017). Additionally, white matter microstructural changes increased with time since pregnancy, demonstrating a trajectory of decline with a history of preeclampsia but not in women remaining normotensive (Siepmann et al., 2017).

While these studies did not evaluate the presence of WMHs, previously discussed studies suggested that the white matter disruption, including the interstitial fluid mobility, water content abnormalities, demyelination and axonal damage that occur with WMHs (Wardlaw et al., 2015) may play a role in cognitive decline. As damage worsens, more measurable cognitive changes occur. Kynast et al. (2018) examined a broad age range of participants for the presence of WMH with correlations of a neuropsychological assessment and report a small decline in cognition with Fazekas scores of 1-2, but more measurable declines with scores of 3–4. As small WMH load can influence cognitive functioning in cognitively healthy middle-aged adults (Brugulat-Serrat et al., 2020), further evaluation of populations prior to evidence of clinical disease is important to understand the trajectory of neurovascular aging and cognitive decline.

Altered cardiovascular function is also reported to play a role in cognition. In a study of postmenopausal women, worsened executive function performance was associated with worsened aortic hemodynamics in women with a history of preeclampsia but not those with healthy pregnancy outcomes (Miller et al., 2020). While the recognition and identification of WMHs in preclinical populations is an important indicator of future risk and decline, subclinical cardiovascular function also appears to serve as important for identifying preclinical risk of changes within the brain.

Although neither flow mediated vasodilation or white blood cell concentration were statistically significant, these results point to the need for further study as the physiological implications of these findings are intriguing. The increased inflammation observed with higher WBC count in those with WMHs was of interest, as increased WMH burden is observed with inflammatory conditions, including an association with visceral adiposity even after controlling for age, sex, and blood pressure (Lampe et al., 2019). This leads to the need to further evaluate proinflammatory cytokine profiles to try to identify mechanistic pathways for increased inflammation.

The endothelium releases nitric oxide, which plays an important role in cerebral circulation (White et al., 1998; Hoth et al., 2007). In older adults, impaired endothelial function is associated with WMH volume (Hoth et al., 2007). In women of reproductive age, estradiol suppression resulted in reduced endothelial activity, assessed by brachial artery flow-mediated vasodilation; endothelial activity was rescued in with vitamin C supplementation in these women, an experimental approach to enhance oxidation and promote endothelial activity (Moreau et al., 2020). In our study participants, we observed reduced maximum dilation in both the popliteal and brachial arteries of those with WMHs in response to flow-mediated vasodilation. While not statistically significant, our analyses indicated that women with decreased endothelial response to vasodilatory challenge were more likely to have WMHs than those with more robust response. The clinical implications of this observation suggest this impairment of peripheral vascular function may indicate reduced endothelial responsiveness throughout the body, including within the brain.

As this was a small study, we were not powered to detect small but potentially physiologically important subclinical differences between women with and without WMHs. Additionally, as a cohort study, we were forced to evaluate a single snapshot in participants’ lives, which may not be representative of lifestyle or life events that can impact aging. Several studies have evaluated whether location of WMH lesions plays a role in the impact and type of cognitive decline (Kaskikallio et al., 2019; Brugulat-Serrat et al., 2020; Camerino et al., 2021), but our small sample size and limited time frame prevented this type of detailed analysis. The greatest limitation of this study was the lack of cognitive testing, which would have allowed for greater exploration into the interplay between cardiovascular health, brain health, and function.

These early associations may point to some of the manifestations of identifiable, but subclinical vascular contributions to cognitive impairment and dementia. Subclinical cardiovascular dysfunction was observed in healthy, young women with WMHs compared to those without. These factors may be implicated in the mechanisms of decline within the brain which lead to cognitive impairment and dementia. The identification of WMH and associated CV characteristics may point towards a mechanism of how the cardiovascular system influences neurovascular aging. Early identification of those with risk factors, such as MWH or subclinical cardiovascular disfunction would allow for better clinical guidance, including through lifestyle modifications and enhanced monitoring.

## Data Availability

The original contributions presented in the study are included in the article/Supplementary Materials, further inquiries can be directed to the corresponding author.
